# The Relationship of the Test for Respiratory and Asthma Control in Kids Initial Score on the Prognosis of Pre-school Children With Asthma: A Prospective Cohort Study

**DOI:** 10.3389/fped.2021.690333

**Published:** 2021-06-30

**Authors:** Lu Liu, Jing Zhang, Lei Zhang, Shu-Hua Yuan, Jin-Hong Wu, Ming-Yu Tang, Jian-De Chen, Fen Zhang, Xin-Yi Qi, Yong Yin

**Affiliations:** Department of Respiratory Medicine, Shanghai Children's Medical Center, School of Medicine, Shanghai Jiao Tong University, Shanghai, China

**Keywords:** track, asthma, pre-school-age, prognosis, children

## Abstract

**Objective:** The test for respiratory and asthma control in kids (TRACK) is currently the only standard follow-up tool for children under 5 years of age with asthma. The purpose of this study was to investigate the relationship between the TRACK initial score (Ti) and their prognosis after 6 months of follow-up in pre-schoolers with asthma.

**Design:** A prospective cohort study.

**Methods:** The study included pre-schoolers diagnosed with asthma at the Shanghai Children's Medical Center between January 2019 and June 2020, and follow-up for 6 months. TRACK scores, frequency of wheezing and respiratory infections, number of Emergency Department (ED) visits and treatment regimen were collected. According to the TRACK initial score, the children were divided into “Ti < 60 group” and “Ti ≥ 60 group,” and the two groups were compared in terms of TRACK score related indicators, clinical manifestations and treatment.

**Results:** There are 102 pre-schoolers included in the analysis [78 boys (76.5%) and 24 girls (23.5%); mean (SD) age, 28.05 (11.63) months]. After 6 months of follow-up, the TRACK score was improved in both groups, and the “Ti ≥ 60 group” had a higher score, lower rate of uncontrolled asthma and fewer reassessments were required. There was no difference in the number of wheezing attacks between the two groups in terms of clinical presentation, but the “Ti < 60 group” had more respiratory infections and ED visits. Regarding the use of ICSs, in the “Ti < 60 groups,” the dose of ICSs was higher and reduced slowly, and the dose difference between the two groups began to appear after 5 months of follow-up.

**Conclusion:** TRACK is essential for pre-schoolers with asthma at the time they are diagnosed. In addition, if the TRACK initial score is < 60, the probability of poor prognosis is higher.

## Introduction

Asthma is the most common chronic respiratory disease in childhood, characterized by chronic airway inflammation and reversible airflow limitation. Previous reports have shown that asthma is the most costly respiratory diseases, probably causing a decline in the quality of life, disability, reduced life expectancy and even death in children and adolescents (1, 2). Moreover, the prevalence of asthma has been remarkably increasing in recent years, both in western countries and in Asia. The prevalence of asthma among children aged 0–14 in mainland China has increased from 1.97% (2000) to 3.02% (2010) (3). A meta-analysis showed a significant increase in the overall prevalence of asthma among children in all age groups and regions in mainland China from 1990 to 2020 (4). Moreover, the overall prevalence rate of pre-schoolers (3–6 years) was 4.285%, which was significantly higher than infants (0–2 years) (2.049%) and school-age children (7–14 years) (3.027%) (5). In addition, due to the poor self-management ability of pre-schoolers, they also have a higher incidence of exacerbations, emergency visits and hospitalizations than other age groups (6, 7). More importantly, poor asthma control has a negative impact on their long-term prognosis. A previous study showed that prognosis disparities begin early in children's lives and that these early health risks may have long-term implications (8). In one study about the progression into adulthood for individuals with moderate to severe childhood asthma, only 15% experienced remission and the rest of children still had asthma symptoms of varying degrees (9). Therefore, early identification of children whose asthma symptoms may persist and intervention is very important. More attention should be paid to the management of pre-school children with asthma.

In fact, a multicenter cross-sectional study in China (10) has shown that uncontrolled asthma in children was mainly due to poor adherence to asthma treatment, and especially in pre-schoolers. The reasons for this phenomenon are as follows: (1) it is difficult for caregivers lacking medical knowledge to detect the changes in the condition of children on time; (2) When entering the chronic persistence of asthma, caregivers often have over-estimated the level of asthma control they had achieved and thus discontinue treatment (10); (3) Because pre-schoolers are too young to complete pulmonary function examination and even do not cooperate with physical examination, the diagnosis and assessment of asthma by clinicians largely come from the medical history information provided by their caregivers, but the information has greatly deviated from the actual situation. Over the past few years, several tests have been proposed to better address these problems, including “the Test for Respiratory and Asthma Control in Kids (TRACK),” designed explicitly for pre-schooler children under 5 years of age.

TRACK was designed by Murphy et al. (11) in 2007 for pre-schoolers with asthma under 5 years of age and is a simple and efficient tool for assessing asthma control. This caregiver-reported test contains 5 items, covering the risk and impairment domains. Scores for various items ranged between 0 and 20, and the total score of the TRACK was 100. The reliability of TRACK was >0.7 in both development and validation samples, and TRACK displayed a good area under the receiver operating characteristic (ROC) curve based on the NAEPP-based evaluation of asthma control (11). As the optimal cut-off point, a score of 80 was used to distinguish between patients with controlled and uncontrolled symptoms (11), and when the TRACK score changes more than 10 points, we should re-evaluate the procedure for asthma management (12). TRACK is very useful in identifying young children with respiratory control problems, which have been translated into various languages, including Chinese (13), Spanish (14), Korean (15), etc., and those versions have high reliability and validity. However, the significance of TRACK in determining prognosis and guiding asthma management has not been fully established.

The current study aimed to explore the relationship between the initial TRACK score and the prognosis of pre-school children with asthma to play a significant role in prognosis assessment and management plan formulation, and more feasible in clinical practice.

## Method

### Study Design and Setting

This prospective, observational, longitudinal study was conducted from January 2019 to June 2020 at the Shanghai Children's Medical Center (Shanghai). The hospital staff contributing to this trial received systematic training prior to patient enrolment. This study was approved by the ethics committee (Approval No. SCMCIRB-K2016052-1). All caregivers provided written informed consent.

### Study Population

Caregivers of children with asthma were invited and visited the study site, and a brief description of the study was provided to them. Interested caregivers were screened for study inclusion. The inclusion criteria were as follows: (1) the child was an outpatient ≤ 4.5 years of age, regardless of gender; (2) the child was diagnosed with asthma according to GINA standard in our hospital [a history of three or more times wheezing attack per year; exercise-induced, laughing-induced or crying-induced wheezing or coughing; clinical improvement with 2–3 months of regular low-dose inhaled corticosteroids (ICSs) and symptom worsening after ICSs cessation], began regular treatment with ICSs, and was followed up in the outpatient clinic for more than 6 months; (3) Completed the Chinese version of TRACK for more than 6 months on time, and recorded the number of wheezing attack, respiratory infections and ED visits per month; (4) the child's parent or guardian provided written consent and (5) the caregiver had access to a smartphone. The exclusion criteria were as follows: (1) no outpatient follow-up or absence of follow-up data; (2) there are obvious outlier values. For example, the result of TRACK score differs greatly from that of GINA score in the same month; (3) the child had a previous allergic reaction to the ICSs; (4) the child had other respiratory diseases, such as bronchopulmonary dysplasia or occlusive bronchiolitis, or had other systemic diseases such as congenital heart disease or gastroesophageal reflux; (5) the caregiver of the child had a history of psychiatric disease, intellectual deficiency, poor motivation, substance abuse, or other conditions that could limit the validity of informed consent; and (6) the child was involved in a similar study in the past 3 months.

First, we determined that the caregivers included in the study had certain Chinese reading and writing skills. Then, they were trained to use the study application (APP) installed on their smartphones before the follow-up. During the 6 months of follow-up, we monitored whether the caregivers completed the TRACK report and reminded the user to complete the monthly report to ensure compliance.

### TRACK

The test used in this study is the TRACK, a Likert scale. The test measured the condition of asthma control in pre-schoolers using five questions: (1) the frequency of respiratory problems (such as wheezing, coughing and shortness of breath) in the last 4 weeks; (2) frequency of waking up at night due to respiratory problems in the last 4 weeks; (3)frequency of interference of respiratory problems with the child's activities in the last 4 weeks; (4) frequency of using emergency medication in the preceding 3 months; and (5) frequency of high dose of ICSs or systemic corticosteroids use in the past 12 months. Scores for various items ranged between 0 and 20, and the total score of the TRACK was 100.

### Data Collection

After informed consent was obtained, caregivers completed the initial score of TRACK under the guidance of a senior respiratory physician when the child was first diagnosed with asthma and started the treatment. During the follow-up for the next 6 months, the caregivers were prompted to complete TRACK through an app on their smartphone and record the number of wheezing attacks respiratory infections and ED visits per month. The treatment plan and medication use of pre-schoolers with asthma were determined by a senior respiratory physician and recorded in the electronic medical records. At the end of the 6-month follow-up, the asthma control levels of the preschoolers were evaluated by physicians based on the GINA assessment for children under 5 years of age.

### Statistical Analysis

SPSS V. 26.0 (IBM SPSS Statistics, USA) was used for the statistical analyses. Kolmogorov-Smirnov-test was used to examine the normality of the data distribution. Measurement data consistent with the normal distribution were expressed as Mean ± SD and an independent sample *t*-test was used for inter-group comparison. The measurement data that did not conform to the normal distribution were represented by the median (quartile spacing) [M (Q1, Q2)], and the Mann-Whitney U test was used for the comparison between groups. Enumeration data were expressed as a percentage (%), and the χ^2^-test was used for inter-group comparison. The accuracy of Ti for predicting individuals asthma control levels (according to the GINA assessment) was assessed by ROC curve analysis. Generalized estimating equations were used to estimate the group population mean for each outcome over time to control the correlation among longitudinal measures within an individual. For binary and continuous outcomes, binomial logistic and normal distributions were specified, respectively. In addition, Kaplan-Meier method was used to detect the reduction rate of controller medications. Two-sided *P* < 0.05 indicated statistical significance.

## Results

### Demographics

**Figure 1:**
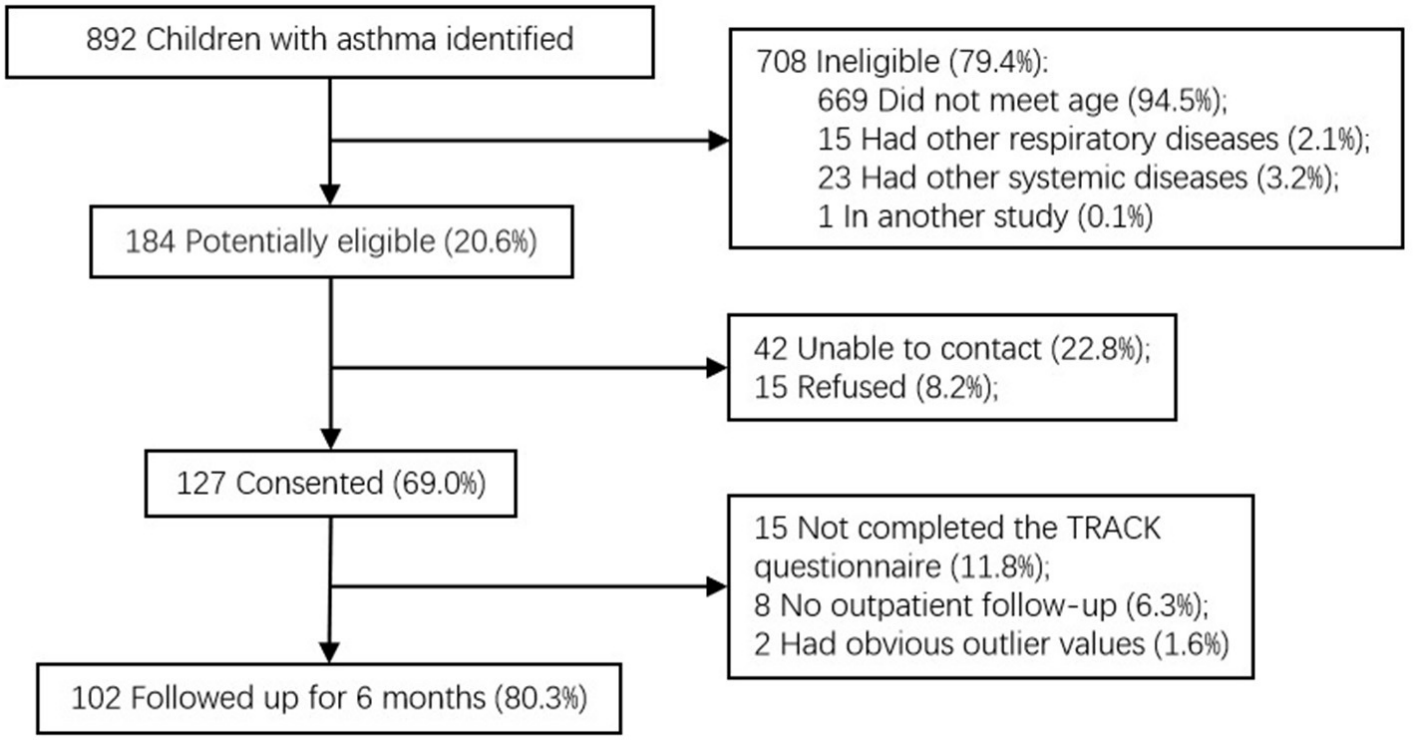
Flow diagram for the selection of participants.

**Figure 2:**
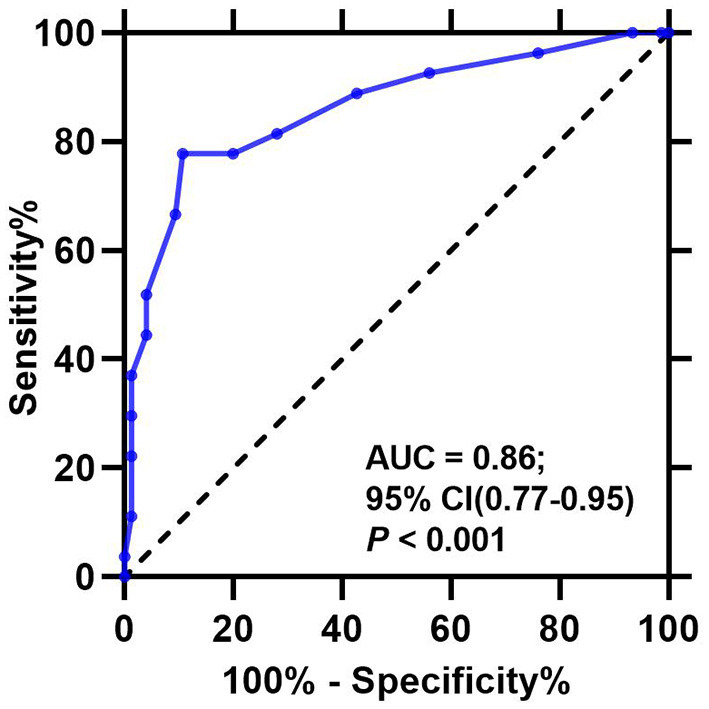
Receiver operating characteristic (ROC) curve for the Test for Respiratory and Asthma Control in Kids (TRACK) initial scores. AUC, area under the curve.

A total of 127 caregivers (for 69.0% of children with asthma potentially eligible) consented to take part in the study. Among them, 102 (80.3%) caregivers completed the follow-up visit, and their TRACK reports were finally evaluated (Figure 1). The TRACK initial score was named as “Ti,” and the subsequent 6-month follow-up TRACK scores were named as “T1, T2, T3, T4, T5, and T6.” According to the results of the GINA assessment, “poorly controlled asthma” was defined as partially controlled and uncontrolled. The receiver operating characteristic (ROC) curve was used to analyze the significance of Ti in measuring the prognosis of asthma. Ti of 62.5 (sensitivity, 89.3%; specificity, 77.8%) were suggested from ROC curve analysis as the best cut-off for differentiating subjects with prognosis (an AUC of 0.86; 95% CI: 0.77–0.95; *P* <0.001; Figure 2). According to this, children with asthma were divided into 2 groups: “Ti <60 Group” and “Ti ≥ 60 Group.” Demographic characteristics are shown in Table 1, and there were no significant group differences in any demographic characteristic.

**Table 1:** Demographic characteristics.

**Characteristics**	**Study group**		
	**All (*n* = 102)**	**Ti <60 (*n* = 25)**	**Ti ≥ 60 (*n* = 77)**
Months of age (months)	28.05 ± 11.63	26.40 ± 12.58	28.58 ± 11.33
**Sex** ***n*** **(%)**			
Male	78 (76.5)	22 (88.0)	56 (72.7)
Female	24 (23.5)	3 (12.0)	21 (27.3)
High (cm)^*^	90.0 (83.0, 97.0)	90.0 (80.0, 95.3)	92.0 (84.5, 97.0)
Weight (kg)^*^	13.72 ± 2.74	13.74 ± 3.12	13.72 ± 2.63
Atopic dermatitis *n* (%)	72 (70.59)	20 (80.00)	52 (67.53)
Allergy rhinitis *n* (%)	62 (60.78)	19 (76.00)	43 (55.84)
Age at first wheezing episode^*^ (months)	12 (6, 18)	9 (6, 18)	14 (6.5, 18)
**Control rating according to the GINA assessment at 6th month of follow-up**			
Controlled *n* (%)	78 (76.47)	9 (36.00)	69 (89.61)
Partly controlled *n* (%)	22 (21.57)	14 (56.00)	8 (10.39)
Uncontrolled *n* (%)	2 (1.96)	2 (8.00)	0 (0)

*Ti, TRACK initial score*.

* *Median and quartiles [median (25, 75%)]*.

### TRACK Score

**Figure 3:**
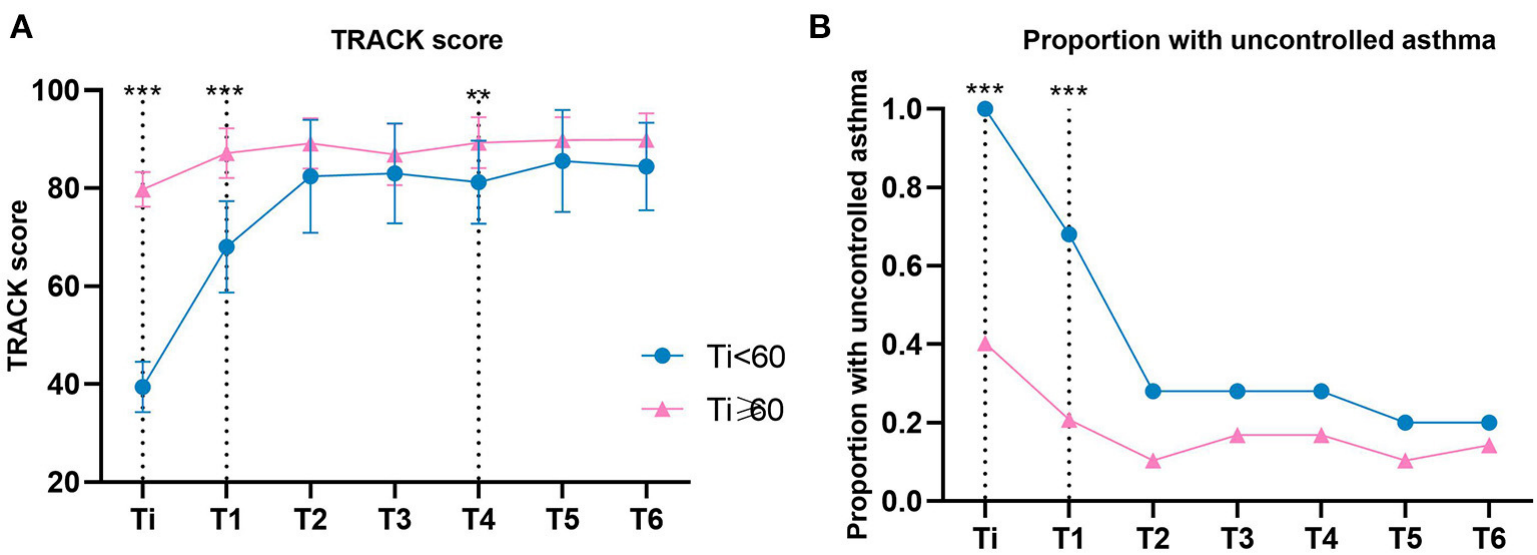
TRACK Score and Asthma Control by Group. **(A, B)** Data were calculated using a generalized estimating equation model adjusted for month. **(B)** Uncontrolled asthma was defined as Track score < 80. Error bars indicate 95% CIs. Ti, TRACK initial score. ***P < 0.001; **P < 0.01.

As shown in Table 2 and Figure 3A, there was an overall improvement in TRACK score for both groups over time, which also represented an improvement in asthma control (Ti <60 group: *P* < 0.001, Ti ≥ 60 group: *P* < 0.001). Meanwhile, there was also a significant difference by time interaction for the Ti < 60 group at 1 and 4 months compared with the Ti ≥ 60 group (T1: *P* < 0.001; T4: *P* = 0.005). In addition, a TRACK score <80 was considered uncontrolled asthma. During 6 months of follow-up, the rate of uncontrolled asthma decreased in both groups (Ti < 60 group: *P* < 0.001, Ti ≥ 60 group: *P* < 0.001), and a significantly lower proportion of pre-schoolers had uncontrolled asthma in the Ti ≥ 60 group at 1 month than the Ti < 60 group (*P* < 0.001) (Table 2 and Figure 3B).

**Table 2:** Description statistics of outcome variables.

**Variable**	**Study group**		
	**All (*n* = 102)**	**Ti < 60 (*n* = 25)**	**Ti ≥ 60 (*n* = 77)**
**TRACK score, median (IQR)**			
Ti^*^	75 (58.75, 85)	45 (25, 55)	80 (75, 87.5)
T1^*^	85 (75, 95)	70 (60, 80)	90 (80, 95)
T2^*^	90 (80, 100)	85 (67.5, 97.5)	90 (85, 100)
T3^*^	90 (80, 100)	80 (72.5, 97.5)	90 (80, 100)
T4^*^	90 (80, 100)	80 (72.5, 90)	95 (85, 100)
T5^*^	90 (80, 100)	90 (80, 95)	95 (82.5, 100)
T6^*^	90 (80, 100)	85 (80, 95)	95 (82.5, 100)
**Uncontrolled asthma No./total No. (%)**			
Ti	56/102 (54.9)	25/25 (100.0)	31/77 (40.3)
T1	33/102 (32.4)	17/25 (68.0)	16/77 (20.8)
T2	15/102 (14.7)	7/25 (28.0)	8/77 (10.4)
T3	20/102 (19.6)	7/25 (28.0)	13/77 (16.9)
T4	20/102 (19.6)	7/25 (28.0)	13/77 (16.9)
T5	13/102 (12.7)	5/25 (20.0)	8/77 (10.4)
T6	16/102 (15.7)	5/25 (20.0)	11/77 (14.3)

*Ti, TRACK initial score*.

* *Median and quartiles [median (25, 75%)]*.

**Figure 4:**
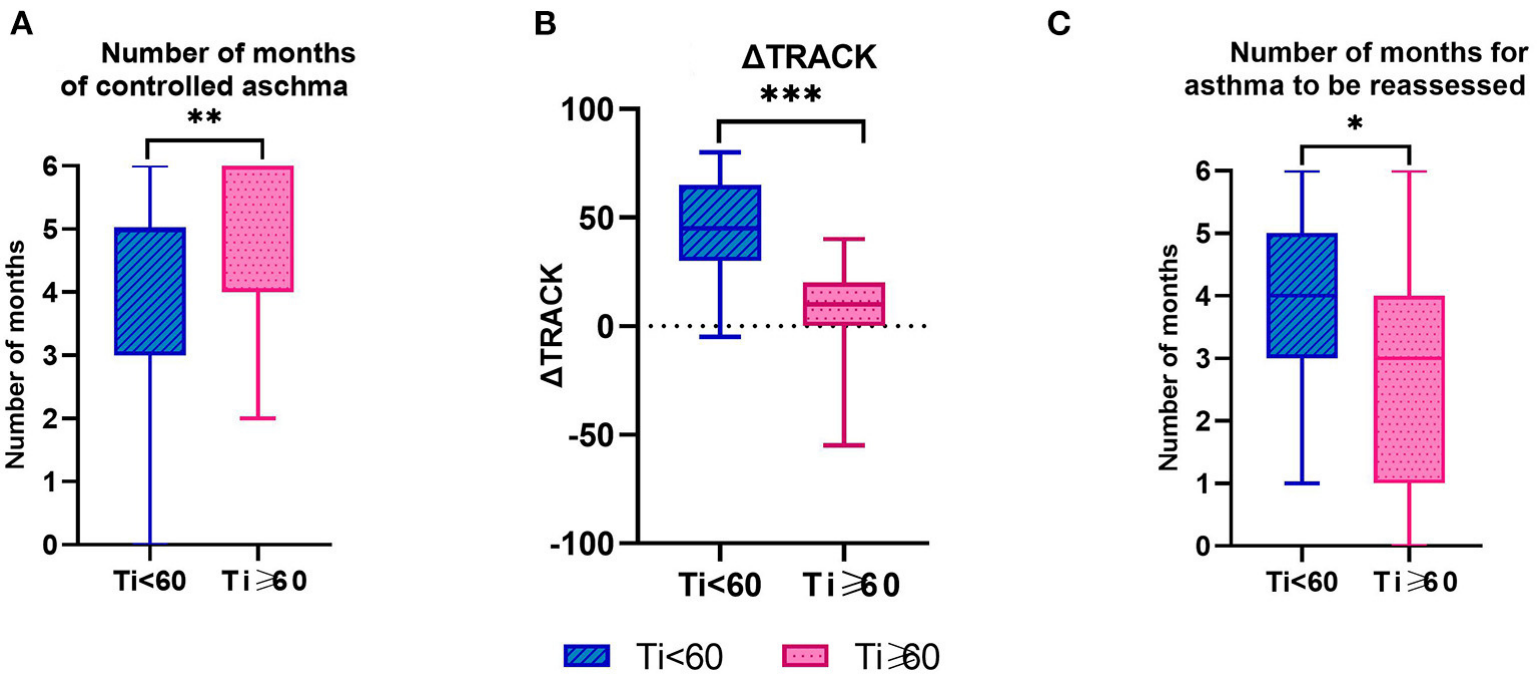
TRACK Score Related Characteristics of Pre-schoolers with Asthma by Groups. **(A)** Controlled asthma was defined as Track score ≥ 80. **(B)** ΔTRACK was defined as T6 minus Ti. **(C)** Asthma should be reassessed when the TRACK score changes by more than 10 points. Ti, TRACK initial score. ***P < 0.001; **P < 0.01; *P < 0.05.

After 6 months of follow-up, the two groups showed different characteristics, which are listed in Table 3. TRACK score ≥ 80 was considered as controlled asthma. The number of months of controlled asthma in the follow-up period of 6 months in the two groups was counted, respectively. There are differences in the number of months of controlled asthma between the two groups (*P* = 0.003, Table 3), and this number in the Ti ≥ 60 group was higher than the Ti < 60 group in the 6 months of follow-up (Figure 4A). ΔTRACK was defined as T6 minus Ti, which was used to measure the variation of TRACK score of pre-schoolers with asthma after 6 months of follow-up. There was a significant difference of ΔTRACK in 2 groups (*P* < 0.001), and after 6 months of follow-up and treatment, the Ti < 60 group has an obviously higher Δ TRACK, which also showed improvement in TRACK score (Table 3 and Figure 4B). Previous relevant studies have shown that asthma should be reassessed when the TRACK score changes by more than 10 points in adjacent 2 months. Based on this principle, we, respectively, calculated the change value of TRACK in adjacent 2 months, and counted the number of months in which the change amount was more than 10 points, which also meant that the number of months needed to be re-evaluated in the 6 months of follow-up. There was a significant difference in the 2 groups (*P* = 0.019), and the number of months that asthma reassessment needs to be conducted is more in Ti < 60 group than in Ti ≥ 60 group (Table 3 and Figure 4C). Meanwhile, it was clearly showed in Table 3 and Figure 3A that the Ti < 60 group improved more quickly in the first few months after diagnosis, it also supports the need for reassessments.

**Table 3:** Characteristics of pre-schoolers with asthma at 6-mouth follow-up visit.

**Variable**	**Study group**		
	**Ti < 60 (*n* = 25)**	**Ti ≥ 60 (*n* = 77)**	***P-*value**
**TRACK score related.median (IQR)**
Number of months of asthma control^*^	5 (3.5)	6 (4.6)	0.003
ΔTRACK^*^	45 (30.65)	10 (0.20)	< 0.001
Number of mouths for asthma to be reassessed^*^	4 (3.5)	3 (1.4)	0.019
**ED Visits, median (IQR)**			
The total number of ED visits^*^	0 (0.1)	1 (0.5.2)	< 0.001
**Average number of ED visits, median (IQR)**			
1^*^	0 (0.1)	0 (0.0)	0.006
3^*^	0 (0.1)	0 (0.0)	0.004
6^*^	0 (0.0.5)	0 (0.0)	0.001

*Ti, TRACK initial score; ED, Emergency Department*.

* *Median and quartiles [median (25, 75%)]*.

### Clinical Manifestation

**Figure 5:**
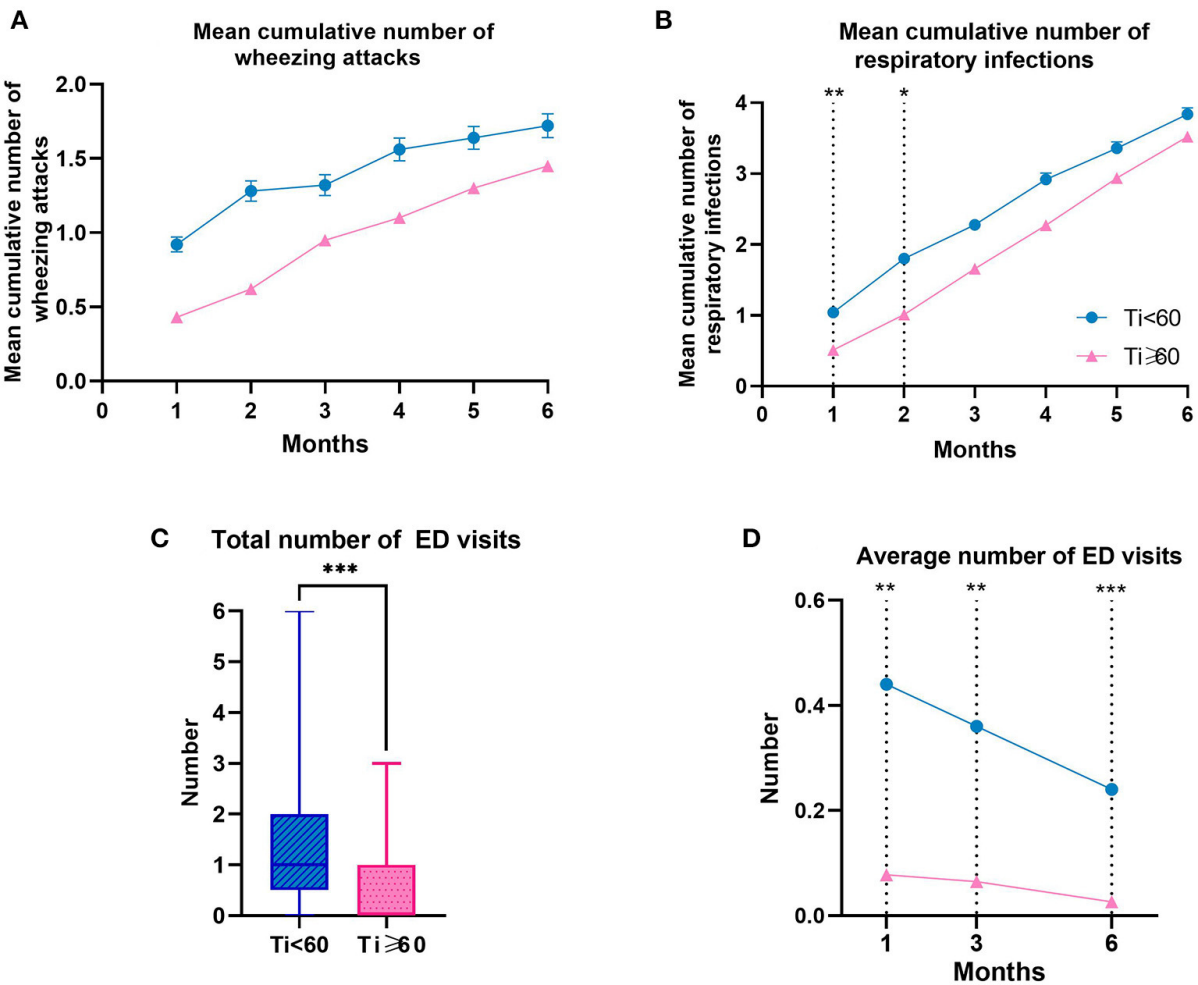
Clinical Manifestation Related Characteristics and the Number of ED Visits of Pre-schoolers with Asthma by Groups. **(A, B)** Data were calculated using a generalized estimating equation model adjusted for month. The data are presented as mean ± SEM. Ti, TRACK initial score. ED, Emergency Department. ***P < 0.001, **P < 0.01, *P < 0.05.

There was no statistical difference in the cumulative mean number of wheezing attacks either in Ti < 60 group or in Ti ≥ 60 group (Table 4 and Figure 5A). As for the mean cumulative number of respiration infections, pre-schoolers in the Ti < 60 group were significantly higher likely to have respiration infections at 1 and 2 months of follow-up compared with the Ti ≥ 60 group (1: *P* = 0.007; 2: *P* = 0.027) (Table 4 and Figure 5B). There was no significant difference among groups at months 3, 4, 5, and 6 months. As for ED visits, during the 6-month follow-up, pre-schoolers in the Ti < 60 group had more number of ED visits than those in the Ti ≥ 60 group (*P* < 0.001, Table 3 and Figure 5C). In addition, the average number of ED visits decreased over time in both groups, and the average number of ED visits in Ti < 60 group was higher than that in the Ti ≥ 60 group at the 1st, 3rd, 6th month (1: *P* = 0.006; 2: *P* = 0.004; 3: *P* = 0.001) (Table 3 and Figure 5D).

**Table 4:** Description statistics of outcome variables.

**Variable**	**Study group**		
	**All (*n* = 102)**	**Ti < 60 (*n* = 25)**	**Ti ≥ 60 (*n* = 77)**
**Mean cumulative number of wheezing attacks, median (IQR)**			
1^*^	0 (0.1)	0 (0, 1.5)	0 (0.1)
2^*^	0 (0.1)	1 (0.2)	0 (0.1)
3^*^	0.5 (0.2)	1 (0.2)	0 (0.1)
4^*^	1 (0.2)	1 (0.2)	1 (0.2)
5^*^	1 (0.2)	1 (0.3)	1 (0.2)
6^*^	1 (0.2)	1 (0.3)	1 (0.2)
**Mean cumulative number respiration infections, median (IQR)**			
1^*^	0.5 (0.1)	1 (0.5, 1)	0 (0.1)
2^*^	1 (0.2)	2 (1.2)	1 (0.2)
3^*^	2 (1.3)	2 (1.3)	2 (1, 2.5)
4^*^	3 (1.3)	3 (1.4)	2 (1.3)
5^*^	3 (2.4)	3 (2.5)	3 (1.5, 4)
6^*^	4 (2.5)	4 (2.5)	4 (2.5)

*Ti, TRACK initial score*.

* *Median and quartiles [median (25, 75%)]*.

### Use of Inhaled Corticosteroids

**Figure 6:**
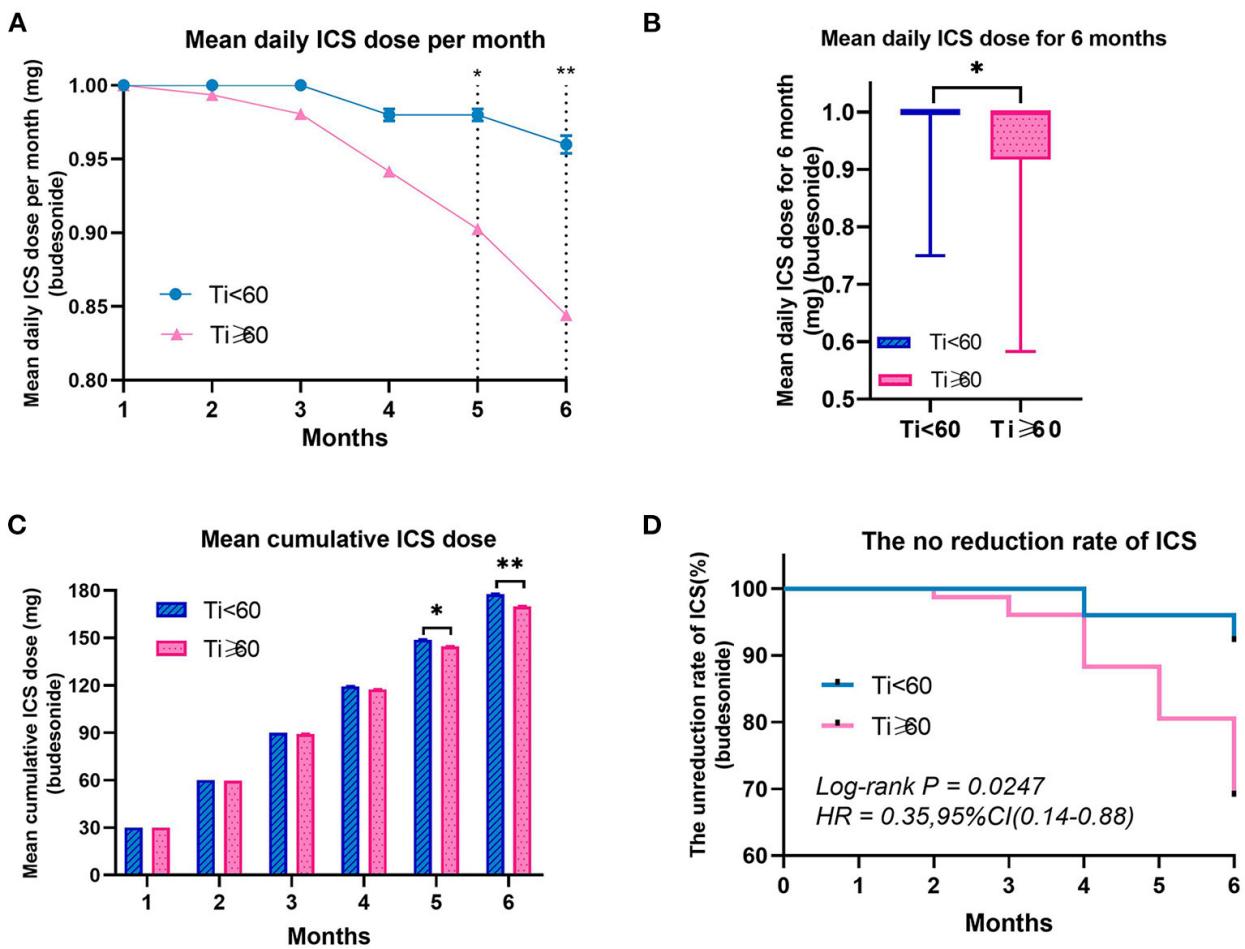
Use of Inhaled Corticosteroids Related Characteristics of Pre-schoolers with Asthma by Groups. **(A, C)** Data were calculated using a generalized estimating equation model adjusted for month. The data are presented as mean ± SEM. ICSs indicate inhaled corticosteroids. Ti, TRACK initial score. **P < 0.01; *P < 0.05.

To visualize prescribed anti-inflammatory medication for asthma in the whole study population, the mean daily ICSs dose per month, mean daily dose for 6 months, mean cumulative dose per month and the un-reduction rate were calculated (Table 5 and Figure 6). There was an obvious decrease in the mean daily ICSs dose per month in Ti ≥ 60 group over time (*P* < 0.001), but this was not the case in Ti < 60 group (*P* > 0.05) (Table 5 and Figure 6A). In addition, differences in the mean daily ICSs dose per month between the two groups began to appear at 5 and 6 months of follow-up (5: *P* = 0.010; 6: *P* = 0.002). The mean daily ICSs dose for 6 months in Ti ≥ 60 group was lower than the Ti < 60 group in the 6 months of follow-up (*P* = 0.022) (Table 5 and Figure 6B). As for the mean cumulative dose of ICSs, the difference between the two groups also occurred at the 5th and 6th months of follow-up (5: *P* = 0.022; 6: *P* = 0.005) (Table 5 and Figure 6C). In terms of the de-escalation of ICSs, the reduction rate was significantly higher in the Ti ≥ 60 group compared to the Ti < 60 group (*P* = 0.0247) (Table 5 and Figure 6D).

**Table 5:** Description statistics of outcome variables.

**Variable**	**Study group**		
	**All (*n* = 102)**	**Ti < 60 (*n* = 25)**	**Ti ≥ 60 (*n* = 77)**
**Mean daily ICSs dose per month (mcg), median (IQR)**			
1^*^	1,000 (1,000, 1,000)	1,000 (1,000, 1,000)	1,000 (1,000, 1,000)
2^*^	1,000 (1,000, 1,000)	1,000 (1,000, 1,000)	1,000 (1,000, 1,000)
3^*^	1,000 (1,000, 1,000)	1,000 (1,000, 1,000)	1,000 (1,000, 1,000)
4^*^	1,000 (1,000, 1,000)	1,000 (1,000, 1,000)	1,000 (1,000, 1,000)
5^*^	1,000 (1,000, 1,000)	1,000 (1,000, 1,000)	1,000 (1,000, 1,000)
6^*^	1,000 (500, 1,000)	1,000 (1,000, 1,000)	1,000 (500, 1,000)
**Mean cumulative ICSs dose, (mcg), median (IQR)**			
1^*^	30,000 (30,000, 30,000)	30,000 (30,000, 30,000)	30,000 (30,000, 30,000)
2^*^	60,000 (60,000, 60,000)	60,000 (60,000, 60,000)	60,000 (60,000, 60,000)
3^*^	90,000 (90,000, 90,000)	90,000 (90,000, 90,000)	90,000 (90,000, 90,000)
4^*^	120,000 (120,000, 120,000)	120,000 (120,000, 120,000)	120,000 (120,000, 120,000)
5^*^	150,000 (150,000, 150,000)	150,000 (150,000, 150,000)	150,000 (150,000, 150,000)
6^*^	180,000 (180,000, 180,000)	180,000 (180,000, 180,000)	180,000 (165,000, 180,000)

*ICSs, inhaled corticosteroids; Ti, TRACK initial score*.

* *Median and quartiles [median (25, 75%)]*.

## Discussion

Asthma is a common chronic disease of the respiratory system, whose incidence has been increasing year by year in recent years. One of the major challenges in asthma management is poor patient compliance, and this phenomenon is more profound in pre-school children with asthma. The TRACK was developed as a follow-up test specifically for children under 5 years of age, based on the impairment and risk domains of the NAEPP guidelines (11). Previous studies have proved that TRACK has high reliability and effectiveness in both cross-sectional and longitudinal evaluations (11, 16). Moreover, these studies all showed that a cut point score of 80 provided the best discrimination between children with controlled and uncontrolled respiratory symptoms. This study takes the relationship between the TRACK initial score and the prognosis of pre-schoolers with asthma as the starting point, aiming to expand the clinical value of the TRACK score in evaluating the prognosis.

Through a 6-month follow-up, this study recorded the significance of the TRACK initial score in reflecting the prognosis of asthma in pre-school children from the aspects of TRACK-related indicators, clinical manifestations, ED visits and the use of inhaled corticosteroids. Moreover, the demographic characteristics of pre-schoolers with asthma included in this study are similar to those in previous studies, and thus comparable (11–13, 16).

According to the follow-up results of TRACK-related indicators, during the 6 months follow-up, a pre-schooler with a lower TRACK initial score was likely to continue to have a lower TRACK score, higher proportion of uncontrolled asthma, and need to be reassessed more frequently. It is noteworthy that the optimal cut-off point for distinguishing asthma outcomes in pre-schoolers was 60 in this study, which was different from the 80 cut-off point for distinguishing asthma control in previous studies. The reason may be that they measured different objects. TRACK score in this study was used to measure prognosis, which was a prospective prediction. However, the TRACK score in previous studies was an assessment of the current situation, a cross-sectional comparison. A 3-month prospective cohort study conducted by Harel-Sterling et al. showed that 60 was also the best predictor of wheezing exacerbations for pre-schoolers who visited the emergency department (ED) for wheezing (17). The best cut-off point of TRACK score in prognostic prediction should be further discussed in future studies.

As for the differences in clinical manifestations, there was no statistical significance in the influence of TRACK initial score on the number of wheezing attacks. However, in terms of respiratory infections and the number of ED visits, pre-schoolers with lower TRACK initial scores had a higher incidence. Respiratory infection is not only a common inducing factor of asthma attack, but also an important reason for asthma deterioration. The viral infection reportedly accounts for 80–85% of asthma exacerbations (18), and respiratory infections caused by some special viruses [such as a respiratory syncytial virus (RSV), rhinovirus (RV), etc.] are not only early markers of recurrent wheezing, but also lead to decrease in lung function (19). Meanwhile, some studies have shown that the number of early respiratory infections is associated with asthma occurrence and development (20). Past researches have shown that when respiratory tract infection occurs, the pathogen is also an atopic allergen, which can also stimulate the immune response of the body, thus increasing airway inflammation and hyperresponsiveness (21), which may be the reason why respiratory tract infection can lead to poor control of asthma. Our study also demonstrated that children with lower TRACK initial scores might have a smaller improvement in asthma control and were more likely to develop acute attacks, to some extent which confirms the validity of previous studies.

As far as the use of ICSs is concerned, pre-schoolers with lower TRACK initial scores had more likely to use more dose of ICSs and had a lower reduction rate. ICSs are the most effective treatment for childhood asthma and are recommended by many international guidelines for asthma management (22). For children under 5 years of age, long-term ICSs (e.g., budesonide) are the first-line treatment for asthma maintenance, and inhalation is the preferred way (23), as was the treatment regimen for the children in this study. ICSs can improve lung function and reduce symptoms, risk of exacerbations, hospitalizations, and death (24). However, our results showed that during the 6 months follow-up, pre-schoolers with lower TRACK initial scores used more dose of ICSs, but still have more times of respiratory infections, indicating that they require more treatment. Further studies are needed to determine whether the dose of ICSs should be increased further or whether montelukast should be added. Meanwhile, although the therapeutic efficacy of ICSs is well-established, there is substantial heterogeneity in response to ICSs treatment from person to person (25). In the following study, attention should be paid to the response to ICSs of pre-schoolers with lower TRACK initial score.

This study has limitations that warrant consideration. TRACK was originally applicable to children under 5 years old, but to meet the follow-up time of 6 months, the age of the included pre-schoolers was limited to under 4.5 years old, which inadvertently led to the exclusion of some children. In addition, the data in this study came from the application filled by the guardians, and there may be some inevitable errors. This study was a single-center retrospective study with follow-up for 6 months, so it had disadvantages such as short follow-up time and poor randomization, and the included children were from Shanghai, China. Therefore, further studies are needed to generalize the conclusions of this study to other populations.

This study has important implications for clinical practice. TRACK is the only suitable for asthma follow-up of pre-schoolers under 5 years old. It is filled by caregivers and has the characteristics of simplicity and easy management. Studies have shown that differences in the prognosis of children with asthma begin early in life (8), so it is very important to identify children at an early stage. We should include the TRACK as a useful assessment item in the first visit of children with asthma, and make a preliminary prediction of prognosis through the TRACK initial score. Nonetheless, the conclusion of this study requires a larger sample size and a longer time to verify its validity.

## Conclusion

TRACK is indispensable for pre-schoolers who come to the clinic for the first time and are diagnosed with asthma. At follow-up over the next 6 months, pre-schoolers with lower TRACK initial scores continued to have lower TRACK scores, and a higher proportion of them had uncontrolled asthma. Therefore, more time is needed to reevaluate their condition. Meantime, pre-schoolers with lower TRACK initial scores used more dose of ICSs, but still had more frequent respiratory infections and ED visits. In summary, our results suggest that pre-schoolers with lower TRACK initial scores have a relatively poor prognosis (60 maybe a good cutoff value), and more attention should be paid to this group. Further studies are needed to demonstrate the validity of this finding in other populations and develop further management measures for them.

## Data Availability Statement

The original contributions presented in the study are included in the article/supplementary material, further inquiries can be directed to the corresponding authors.

## Ethics Statement

The studies involving human participants were reviewed and approved by Ethics Committee of Shanghai Children's Medical Center Affiliated to Shanghai Jiao Tong University School of Medicine. Written informed consent to participate in this study was provided by the participants' legal guardian/next of kin.

## Author Contributions

X-YQ, YY, and LL contributed to the conception and design of the study and revising the draft critically for important intellectual content. YY and LL contributed to data analysis and contributed to drafting the submitted article. LL, JZ, LZ, S-HY, J-HW, M-YT, J-DC, and FZ contributed to the data acquisition and interpretation of the outcomes. X-YQ and YY contributed to crucial revision of the draft for important intellectual content and providing final confirmation of the revised version to be published. All authors contributed to data analysis, drafting the manuscript, amending the paper and being responsible for all aspects of the work. All the data could be accessed to all of the authors assured the accuracy of the reported data.

## Conflict of Interest

The authors declare that the research was conducted in the absence of any commercial or financial relationships that could be construed as a potential conflict of interest.
